# A Case Report of Pulmonary Sarcoidosis: An Uncommon Cause of Chest Pain

**DOI:** 10.5811/cpcem.2020.7.48310

**Published:** 2020-09-09

**Authors:** Justina Truong, John Ashurst

**Affiliations:** Kingman Regional Medical Center, Department of Emergency Medicine, Kingman, Arizona

**Keywords:** Chest pain, pulmonary sarcoidosis, sarcoidosis

## Abstract

**Introduction:**

Chest pain is one of the most common causes of emergency department visits on an annual basis and carries a high degree of morbidity and mortality if managed inappropriately.

**Case Report:**

A 36-year-old male presented with four months of left-sided chest pain with dyspnea on exertion. Physical examination and laboratory values were within normal limits. Chest radiograph depicted diffuse interstitial nodular opacities throughout the lungs bilaterally with bilateral perihilar consolidations. Computed tomography of the chest demonstrated mid and upper lung nodularity with a perilymphatic distribution involving the central peribronchial vascular regions as well as subpleural and fissural surfaces causing conglomerate in the upper lobes centrally with associated hilar and mediastinal lymphadenopathy. The next day the patient underwent bronchoscopy with endotracheal ultrasound and transbronchial biopsies and pathology revealed non-necrotizing, well-formed granulomas embedded in dense hyaline sclerosis consistent with sarcoidosis.

**Discussion:**

Sarcoidosis is a multi-system granulomatous disease characterized by noncaseating granulomas on pathology. The worldwide epidemiology of sarcoidosis is currently unknown due to many patients being asymptomatic. However, patients may present with a persistent cough, dyspnea, or chest pain. Emergency department management should be aimed at minimizing long-term sequelae of the disease through obtaining labs and imaging after specialist consultation and arranging urgent follow-up.

**Conclusion:**

Although not one of the six high-risk causes of chest pain, sarcoidosis should be included in the differential to minimize the risk of long-term morbidity associated with advanced forms of the disease.

## INTRODUCTION

Chest pain is the second most common complaint seen by emergency care providers and accounts for 6.4 million visits annually.^1^ Emergency clinicians must be able to differentiate the different causes of chest pain in order to minimize both acute and long-term morbidity and mortality. Although not normally a cause of short-term morbidity or mortality, pulmonary sarcoidosis should be considered as an acute cause of chest pain in the correct patient population and should be managed aggressively to prevent long-term complications from the disease. We present a case of pulmonary sarcoidosis in a 36-year-old male that was managed with urgent consultations and procedures to minimize his risk of long-term complications.

## CASE REPORT

A 36-year-old White male presented to the emergency department (ED) with a four-month history of left-sided chest pain with associated shortness of breath on exertion. He described the pain as a dull ache that occurred at rest and was not worsened by exertion. He also noted a chronic dry cough that he had for the prior several years that was not associated with illness or exercise and a 12-pound weight loss over the previous month. Past medical history was noted for ureterolithiasis several years prior and he took no medications on a daily basis. His last purified protein derivative skin test was several months prior and was negative. He also denied ever smoking or a family history of autoimmune or inheritable disorders.

Upon arrival, his vital signs were all within normal limits and his examination exhibited only scant wheezes and coarse breath sounds in the left upper lobe. Egophony and whispered pectoriloquy were both negative in the concerned area. He also had no discernible skin lesions or clubbing.

**Image 1:**
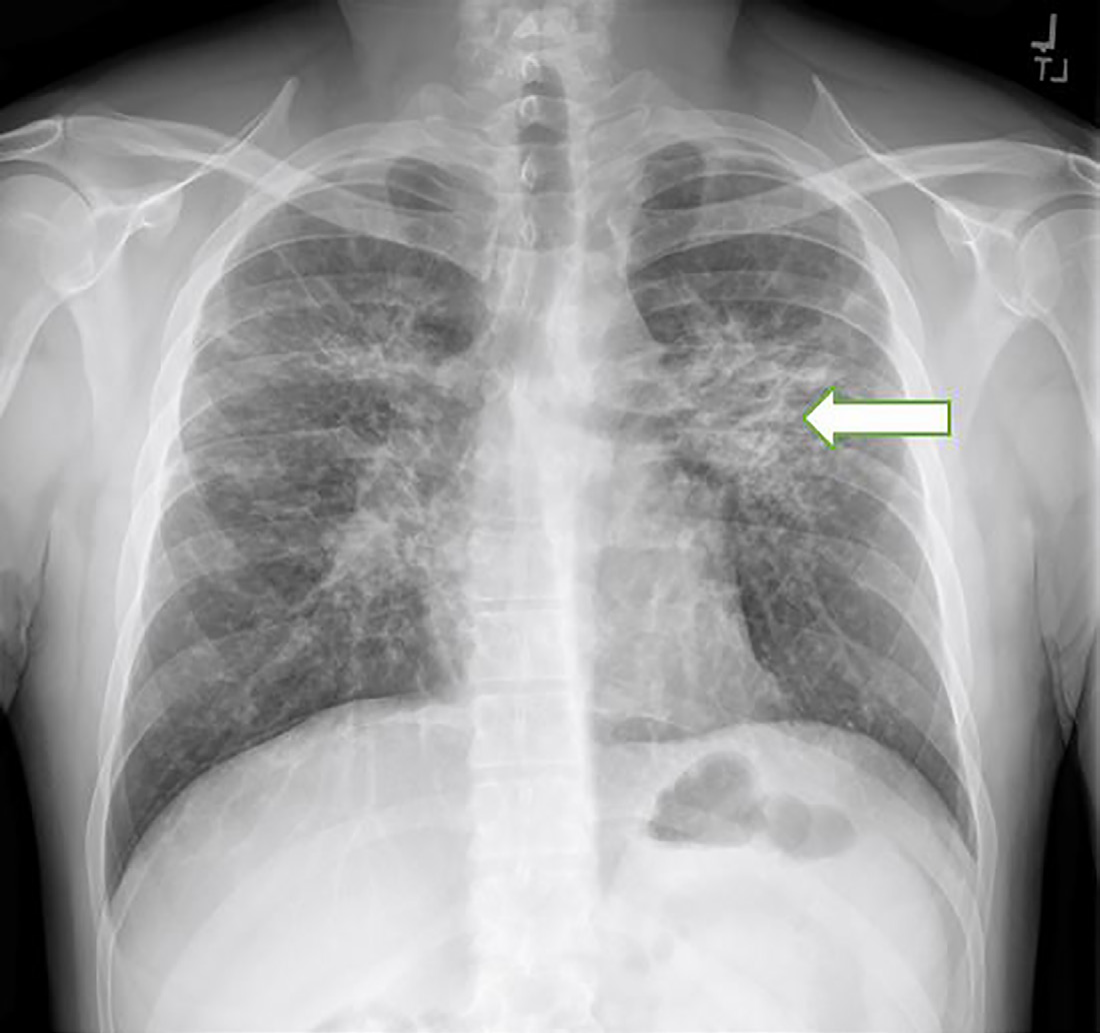
Chest radiograph depicting diffuse interstitial nodular opacities throughout the lungs bilaterally with bilateral perihilar consolidations that were worse on the left (arrow).

**Image 2:**
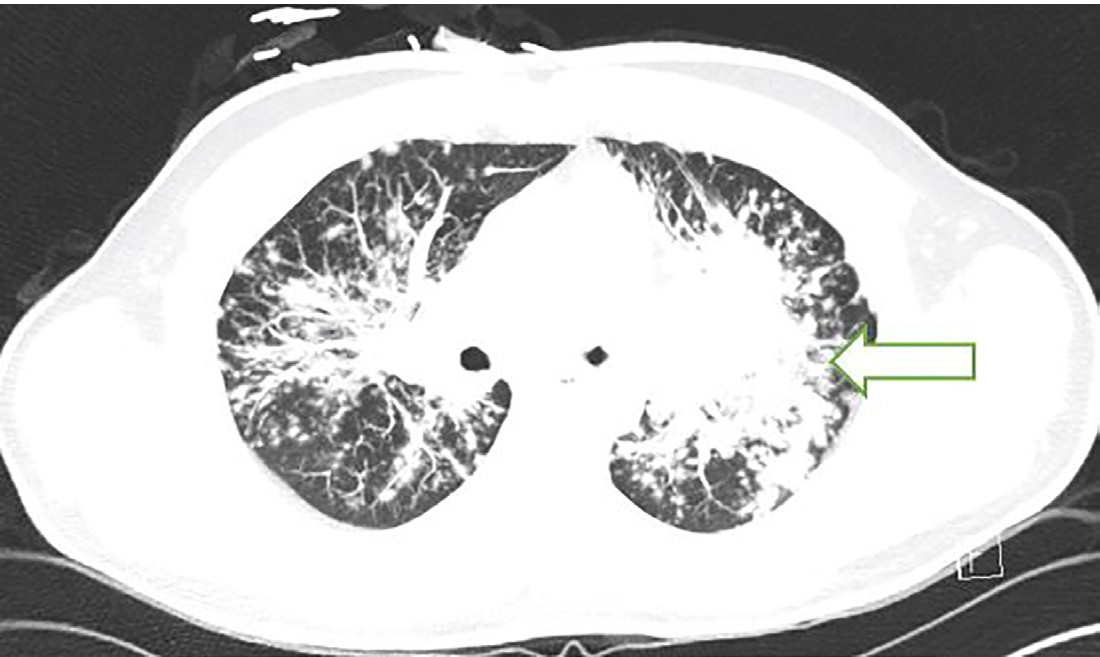
Computed tomography of the chest with intravenous contrast depicting mid and upper lung nodularity with a perilymphatic distribution involving the central peribronchial vascular regions as well as subpleural and fissural surfaces (arrow).

Electrocardiogram showed a normal sinus rhythm with 82 beats per minute without any signs of ischemia. Laboratory testing including a complete blood count, complete metabolic profile, and troponin T test were all negative. Chest radiograph (CXR) demonstrated diffuse interstitial nodular opacities throughout the lungs bilaterally with bilateral perihilar consolidations that were worse on the left (Image 1). Computed tomography with intravenous contrast of the chest showed mid and upper lung nodularity with a perilymphatic distribution involving the central peribronchial vascular regions as well as subpleural and fissural surfaces causing conglomerate in the upper lobes centrally (Image 2). There was also mild symmetric bilateral hilar and mediastinal lymphadenopathy.

CPC-EM Capsule

What do we already know about this clinical entity?

*Sarcoidosis is a multi-system granulomatous disease characterized by noncaseating granulomas that can acutely present with chest pain or dyspnea*.

What makes this presentation of disease reportable?

*Pulmonary sarcoidosis is an uncommon cause of chest pain seen by the emergency physician*.

What is the major learning point?

*Emergency department management should be aimed at specialty consultation with agreed upon follow-up to prevent long term sequelae*.

How might this improve emergency medicine practice?

*Although not one of the six high-risk causes of chest pain, pulmonary sarcoidosis should be in the differential to minimize the risk of long-term morbidity*.

After discussion with pulmonology, the differential included lymphoma, tuberculosis, fungal infections, and pulmonary sarcoidosis. The following day, the patient underwent bronchoscopy with endotracheal ultrasound and transbronchial biopsies. Bronchoalveolar lavage was negative for fungal infections, acid-fast bacilli, and malignant cells. Endobronchial biopsies revealed numerous non-necrotizing, well-formed granulomas embedded in dense hyaline sclerosis.

The patient was subsequently diagnosed with stage 3 pulmonary sarcoidosis and started on prednisone daily and sulfamethoxazole/trimethoprim three times a week for eight weeks. Following treatment, he had resolution in his symptoms.

## DISCUSSION

Sarcoidosis is a multi-system, granulomatous disease without a known etiology. However, it is characterized by a T-helper cell response to CD-4 lymphocytes and activated macrophages that accumulate in the affected organs.^2^ Most studies suggest that the pathogenesis is related to an exaggerated immune response to an environmental factor, microbe, or antigen in a genetically susceptible individual.^3^

Although the worldwide epidemiology of sarcoidosis is difficult to ascertain due to a large proportion of patients being asymptomatic, it has been estimated that 60 out of 100,000 adults in the United States will be affected by the disease.^4^ More than 80% of these cases will be diagnosed between the ages of 20–50 with a second peak in incidence between 50–65 years of life.^2^,^3^ Females, nonsmokers, and Blacks are more commonly diagnosed with the disease and 10% of cases will be familial.^2^–^4^ Mortality has been estimated at between 2–5% secondary to pulmonary complications, while morbidity can be substantial due to poor outcomes in chronic sarcoidosis.^5^

The onset and presentation of sarcoidosis varies widely depending on what organ system may be involved; so maintaining suspicion for this diagnosis is crucial. The lungs are the most commonly affected organ in the disease process, with more than 90% of patients exhibiting pulmonary symptoms.^2^ The liver, spleen, bones, skin, heart, and nervous system may also be involved.^2^,^3^ Up to one third of patients are asymptomatic at their time of presentation with findings discovered incidentally.^6^

Clinical presentations most commonly include unexplained or persistent cough, dyspnea, or chest pain.^6^,^7^ Additional symptoms such as fever, erythema nodosum, and hilar lymphadenopathy (known as Löfgren syndrome) may be seen.^3^,^4^ Extrapulmonary findings such as hypercalcemia, nephrolithiasis, arthritis, and heart failure can be present and should warrant further investigation.^6^ Heerfordt-Waldenström syndrome is another classic syndrome of sarcoidosis that includes uveoparotid fever and facial nerve palsies.^2^,^3^

Often, the diagnosis of sarcoidosis is not made until 3–6 months following the initial presentation.^6^ The three criteria for the diagnosis of sarcoidosis include compatible clinical and radiologic findings, histopathologic evidence of noncaseating granulomas, and exclusion of other disease processes.^3^ Laboratory studies should include a complete blood count, a metabolic panel to include creatinine and liver function testing, calcium levels in blood and urine, Vitamin D assays, and an angiotensin-converting enzyme level.^3^ Testing for the human immunodeficiency virus as well as tuberculosis should also be performed.^3^ CXR, computed tomography of the chest, flexible bronchoscopy with biopsies and bronchioalveolar lavage, and pulmonary function testing should be performed in certain cases.^3^ CXR can show findings of lung involvement categorized into four stages based on the Scadding scale (Table).^2^

Treatment for sarcoidosis is recommended for those with active disease and who are symptomatic.^4^ Those with stage I and who are asymptomatic need no treatment but do require annual follow-up.^3^ First-line treatment for those with symptomatic stages II or III is systemic corticosteroids and follow-up every three months.^3^ Corticosteroids are also recommended for those with serious extrapulmonary disease.^3^ Treatment with 20–40 milligrams of prednisone per day for four to six weeks and tapering slowly if condition improves is the mainstay of therapy.^3^ Response to treatment is monitored every three to six months using clinical response, pulmonary function testing, and CXR.^3^,^7^

Methotrexate is the most common second-line agent for treatment of pulmonary sarcoidosis. Azathioprine, leflunomide, and the TNF-alpha inhibitor infliximab are reserved for those who cannot tolerate corticosteroids or those with refractory symptoms and disease progression.^3^,^4^ Those with severe pulmonary disease failing treatment or those progressing to pulmonary fibrosis in stage IV need prompt referral and evaluation for lung transplantation.^3^ Treatment is also aimed at regular screening for and treatment of complications such as cardiac sarcoidosis and heart disease as well as pulmonary hypertension.

Recurrence and relapse are common in those with pulmonary sarcoidosis.^4^ Those undergoing treatment with corticosteroids and immunosuppressive medications are at risk of the adverse effects such as diabetes mellitus, hypertension, and opportunistic infections such as pulmonary aspergillosis and *Pneumocystis jirovecii*.^3^,^6^ The most common cause of sarcoidosis-related death in the United States is respiratory failure related to pulmonary fibrosis.^2^,^3^,^7^ Pulmonary hypertension is likely to develop in those with severe pulmonary fibrosis and chronic forms of the disease, which is in itself an indicator for increased mortality and for lung transplantation.^2^,^6^

## CONCLUSION

Although emergency clinicians are trained in diagnosing and treating the deadly causes of chest pain, they must take one step further when diagnosing a patient with a relatively low-risk disease from an emergency medicine perspective that carries a high degree of long-term morbidity. ED management should be aimed at proper specialty consultation with agreed upon follow-up in the symptomatic patient with consultation to specialties as needed.

## Figures and Tables

**Table:** Scadding scale for staging of pulmonary sarcoidosis.

Stage	Chest radiograph findings
I	Bilateral hilar lymphadenopathy
II	Pulmonary infiltrates and bilateral hilar lymphadenopathy
III	Pulmonary infiltrates
IV	Pulmonary fibrosis
